# Self-Administered Skills-Based Virtual Reality Intervention for Chronic Pain: Randomized Controlled Pilot Study

**DOI:** 10.2196/17293

**Published:** 2020-07-07

**Authors:** Beth D Darnall, Parthasarathy Krishnamurthy, Jeannette Tsuei, Jorge D Minor

**Affiliations:** 1 Department of Anesthesiology, Perioperative and Pain Medicine Stanford University School of Medicine Palo Alto, CA United States; 2 C.T. Bauer College of Business University of Houston Houston, TX United States; 3 AppliedVR, Inc Los Angeles, CA United States; 4 L.A. Pain & Wellness Institute Los Angeles, CA United States

**Keywords:** chronic pain, virtual reality, behavioral medicine, self-management, mobile phone, randomized controlled trial

## Abstract

**Background:**

Patients with chronic pain often have limited access to comprehensive care that includes behavioral pain management strategies. Virtual reality (VR) is an immersive technology and emerging digital behavioral pain therapy with analgesic efficacy for acute pain. We found no scientific literature on skills-based VR behavioral programs for chronic pain populations.

**Objective:**

The primary aim of this study is to evaluate the feasibility of a self-administered VR program that included content and skills informed by evidence-based behavioral treatment for chronic pain. The secondary aim is to determine the preliminary efficacy of the VR program in terms of average pain intensity and pain-related interference with activity, stress, mood, and sleep, and its impact on pain-related cognition and self-efficacy. The tertiary aim was to conduct a randomized controlled trial (RCT) and compare the VR treatment with an audio-only treatment. This comparison isolated the immersive effects of the VR program, thereby informing potential mechanisms of effect.

**Methods:**

We conducted an RCT involving a web-based convenience sample of adults (N=97) aged 18-75 years with self-reported chronic nonmalignant low back pain or fibromyalgia, with an average pain intensity >4 over the past month and chronic pain duration >6 months. Enrolled participants were randomly assigned to 1 of 2 unblinded treatments: (1) VR: a 21-day, skills-based VR program for chronic pain; and (2) audio: an audio-only version of the 21-day VR program. The analytic data set included participants who completed at least 1 of 8 surveys administered during the intervention period: VR (n=39) and audio (n=35).

**Results:**

The VR and audio groups launched a total of 1067 and 1048 sessions, respectively. The majority of VR participants (n=19/25, 76%) reported no nausea or motion sickness. High satisfaction ratings were reported for VR (n=24/29, 83%) and audio (n=26/33, 72%). For VR efficacy, symptom improvement over time was found for each pain variable (all *P*<.001), with results strengthening after 2 weeks. Importantly, significant time×group effects were found in favor of the VR group for average pain intensity (*P*=.04), pain-related inference with activity (*P*=.005), sleep (*P*<.001), mood (*P*<.001), and stress (*P*=.003). For pain catastrophizing and pain self-efficacy, we found a significant declining trend for both treatment groups.

**Conclusions:**

High engagement and satisfaction combined with low levels of adverse effects support the feasibility and acceptability of at-home skills-based VR for chronic pain. A significant reduction in pain outcomes over the course of the 21-day treatment both within the VR group and compared with an audio-only version suggests that VR has the potential to provide enhanced treatment and greater improvement across a range of pain outcomes. These findings provide a foundation for future research on VR behavioral interventions for chronic pain.

## Introduction

### Background

The US Department of Health and Human Services [1,2], the Institute of Medicine [3], the National Institutes of Health, and the Centers for Disease Control and Prevention have called for the integration of evidence-based behavioral medicine strategies into the treatment of acute and chronic pain. These unified recommendations reflect a common understanding that pain is a biopsychosocial experience that requires a comprehensive treatment approach to equip individuals with skills to actively self-manage pain and symptoms [4].

Evidence-based behavioral pain treatments include cognitive behavioral therapy (CBT) for chronic pain [5-7], acceptance and commitment therapy [8], and mindfulness-based stress reduction [5,6]. Treatments typically include didactic content, goal setting, and experiential skills practice within sessions and skills practice between sessions. Effective chronic pain management techniques include biofeedback and relaxation training, with the latter being the mainstay of every effective behavioral treatment for chronic pain [9]. Evidence-based behavioral medicine treatments are commonly delivered individually or in group format, during treatment classes or sessions that are 1 to 2 hours in duration, with a course of treatment lasting 8-11 weeks [7]. Behavioral treatments for chronic pain have been shown to be effective for reducing pain bothersomeness [5,6], symptoms of depression [7,8], and pain-specific cognitive and emotional distress (eg, pain catastrophizing) [5-7], although these treatments have not been effective in reducing pain intensity. Behavioral treatments have also been shown to be effective for improving pain-related self-efficacy or confidence in one’s ability to self-manage pain and engage in meaningful activities [5,6].

Despite the availability of efficacy data for behavioral treatments, multiple barriers may prevent patients from broadly accessing low-risk, integrative, nonpharmacologic pain self-management tools that address the psychosocial aspects of chronic pain [10]. Such barriers may include few skilled local therapists, poor insurance coverage, copayments associated with clinic visits, travel costs, and treatment time [10]. Even when delivered to participants at no cost, in-person behavioral medicine treatments can have poor patient engagement [11], thereby suggesting that new methods of treatment delivery are required to meet the needs of a broad range of patients. Accordingly, research has demonstrated preliminary efficacy for an ultrabrief, single-session, skills-based behavioral treatment class for chronic pain [12] as well as for mobile health teleconference-delivered multisession behavioral pain treatment [13]. Although both options offer new conveniences and possibly expanded access to care, particularly for patients with mobility limitations, the patient remains dependent on a therapist for the delivery of the treatment.

Digital therapeutics offer independent, home-based, on-demand access to behavioral treatment for acute and chronic pain. For instance, a brief, web-based, 90-min, skilled-based pain treatment was shown to reduce postsurgical opioid use in women who underwent surgery for breast cancer [14]. For chronic pain, digital multisession interventions have been shown to be effective [15-17]. Despite these successes, any one modality will not meet the needs of everyone; indeed, even web-based pain treatments that are offered at no cost have <60% engagement rates [14], thus underscoring the need to offer a diverse range of accessible treatment options for chronic pain and, in particular, to identify treatments that may yield superior outcomes.

**Figure 1:**
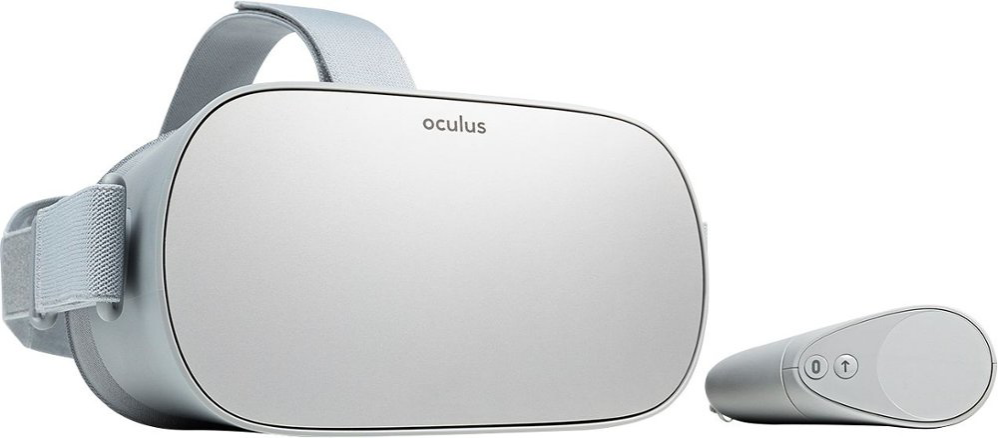
Oculus Go virtual reality headset.

**Figure 2:**
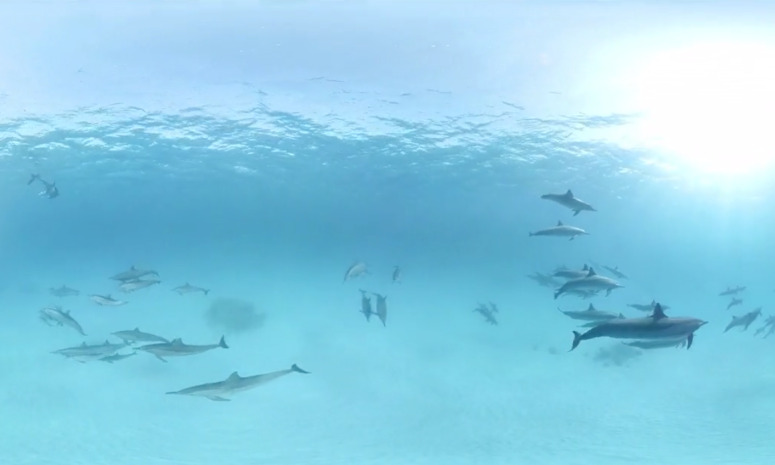
Image of Pain Care virtual reality content (swimming with dolphins).

Virtual reality (VR) has emerged as an effective digital treatment for acute pain. With VR, the user wears a headset that fully restricts the vision field to the content displayed inside the headset screen, and auditory perception is directed to the audio delivered through the device (although not fully restricted; Figure 1). VR provides a multisensory, 3D immersive environment that stimulates the visual, auditory, and proprioception senses, thus engendering the perceptual experience that one is physically located inside and engaged with the virtual environment they are viewing [18,19], such as swimming with dolphins (Figure 2). VR has been used in numerous clinical settings and health conditions to treat anxiety and mental disorders [20-22], aids physical rehabilitation [23,24], and supports postsurgical recovery. Evidence suggests that VR is effective for managing acute pain, including pain elicited during medical procedures [25-29], and burn wound care [30,31] and in hospitalized patients [32,33].

Although several studies have investigated VR for chronic pain, the literature to date is limited. Promising studies have used VR to reduce pain and improve outcomes in complex regional pain syndrome [34], chronic headache/migraine pain [35], fibromyalgia [36,37], and chronic musculoskeletal pain [38,39]. Two recent reviews and meta-analyses reported the efficacy of VR for physical rehabilitation from painful spinal conditions [24] and for orthopedic rehabilitation in terms of reduced pain and disability [40]. In such studies, the user may engage with interactive VR alone or within the context of kinematic training. The literature to date is limited by studies that are conducted in experimental or clinical settings, do not compare to a non-VR group, or include very small samples. Most importantly, the VR studies to date are focused primarily on distraction or physical rehabilitation and do not include pain management education or cultivation of behavioral pain self-management skills (eg, diaphragmatic breathing or cognition and emotion regulation). Crucially, if found to be effectively delivered by VR, such content could serve as either a replacement or treatment extender for in-person behavioral medicine clinic visits. Studies are needed to determine whether behavioral pain management skills-based VR is effective and can facilitate sustained pain relief through skills mastery and increased self-efficacy for pain self-management. The VR platform could transcend many current barriers to care and potentially provide a scalable way to deliver on-demand home-based behavioral treatment for chronic pain.

### Objectives

Building on the nascent literature on VR for chronic pain, we aim to evaluate a skills-based behavioral medicine VR program for chronic low back pain and fibromyalgia to provide preliminary data on its utility and efficacy as a stand-alone home-based treatment for people in the community. To accomplish this goal, we conducted an exploratory randomized controlled trial (RCT) with 3 main aims: (1) to determine the feasibility and satisfaction of a self-administered, at-home, skills-based 21-day VR intervention (pain care VR) for chronic pain; (2) to evaluate the preliminary efficacy of VR intervention for reducing average pain intensity and pain-related outcomes; and (3) to isolate the immersive effects of VR by comparing it with an audio-only treatment group. We hypothesized good feasibility and satisfaction for VR as well as the superiority of VR over audio treatment for pre-post improvement across the pain indicator variables.

## Methods

### Design and Setting

This was a parallel-group, randomized controlled clinical trial involving 2 home-based behavioral interventions applied in a community-based, web-based convenience sample of people with chronic pain conducted between March and May 2019.

### Procedures

Participants were recruited remotely through web-based advertisements on Facebook and The Mighty, a digital health community. Internet and computer literacy were implicit de facto eligibility criteria. Eligibility screening involved a brief telephone assessment for enrollment criteria outlined in the Inclusion and Exclusion Criteria section.

### Inclusion and Exclusion Criteria

Following electronic informed consent (see Multimedia Appendix 1 for the study consent form), study participants were randomized one-to-one using a Research Electronic Data Capture Cloud random number generator and allocated to the treatment group. All study procedures were completed remotely, and no in-person visits were required. Study participants were not blinded to the treatment group assignment because of the obvious nature of the mode of delivery of their assigned treatment. Participants assigned to the VR group were called by a study staff to ensure receipt of the mailed VR kit and for a brief orientation to materials. A study staff member was available by phone at the request of study participants in both groups. Participant compensation was prorated based on the number of surveys completed; they received up to US $30 in the form of an Amazon electronic gift card following completion of their final study survey. The study was approved by the Western institutional review board (Puyallup, WA).

### Treatment Groups

**Figure 3:**
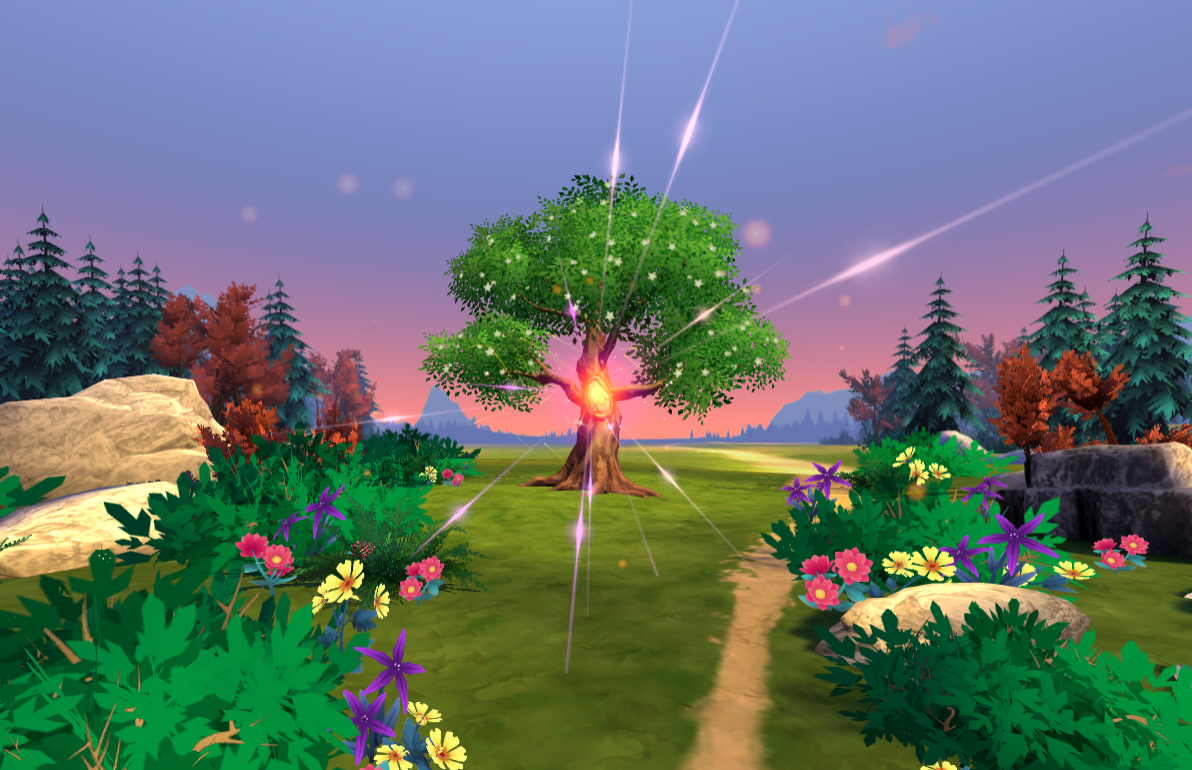
Image of Pain Care virtual reality content that visualizes the user’s breath.

Both treatment groups received the same didactic content delivered in distinct formats (VR versus audio; see Multimedia Appendix 2 for program content). Treatment consisted of a variety of sessions to support participants in learning self-management skills based on evidence-based CBT principles as well as biofeedback and mindfulness strategies used in pain management. The program was designed to improve self-regulation of cognitive, emotional, and physiological responses to stress and pain and comprised 3 main content categories:

- Skills rooted in pain CBT: brief didactics describe how thoughts and emotions can impact pain and techniques on thought restructuring and adaptive regulation of pain-related cognition and emotion (eg, addressing pain catastrophizing tendencies).
- Relaxation training: participants engage in diaphragmatic breathing exercises to enhance parasympathetic nervous system function and decrease physiological hyperarousal. Importantly, relaxation training was optimized in the VR group with visual biofeedback that displayed the environment responding to the users’ physiologic behavior during the exercises (Figure 3). The software uses a patent-pending algorithm to detect the user’s exhalations with the VR headset’s built-in microphone, along with a hardware breath amplifier that directs the user’s breath toward the microphone. The user’s breath is then visualized in the VR environment as either breath particles or waves expanding outward from the user, with the synchronization of physical exhalation and the visual effects deepening the immersion of the experience and helping the user slow and deepen their respiration.
- Mindfulness: mindfulness content encouraged awareness of the mind and body (somatic cues) as well as thought release (nonattachment).

The 21-day program consisted of 4-8 treatment sessions from each content category with the duration of session length ranging from 1 to 15 min. Each treatment session was indexed as complete if the participants initiated the experience. Participants could replay the completed sessions.

#### Virtual Reality Group

VR group participants were mailed an Oculus Go VR headset preloaded with VR software developed by AppliedVR. The Oculus Go is an easy-to-use, stand-alone VR headset with an accompanying orientation-tracked controller, a head-mounted display with a high-resolution screen, built-in spatial audio speakers, and an integrated microphone. A VR user manual sent via email included an instructional video on the proper use of the VR headset. Participants were instructed to telephone VR support staff if they had any questions or difficulty in using the headset. Participants were instructed to complete 1 VR treatment session daily for 21 days (Multimedia Appendix 2).

#### Audio Group

The audio program consisted of the majority of the same narrative content contained in the VR program, with changes made to the descriptive titles for each session. Owing to VR having a visual media form and audio that specifically references the changing images in the VR environment, approximately one-third of the VR program could not be included verbatim in the audio. Rather, the audio session topical content was closely matched to the corresponding VR session for that day and adapted to eliminate any references to visual content that would be confusing to the listener. Participants received an electronic link to the audio recordings on SoundCloud (a music streaming platform) where they could choose to stream or download the audio recordings on their smartphones, laptops, or desktop computers. Participants were instructed to complete 1 audio treatment session daily for 21 days (Multimedia Appendix 2).

### Data Collection and Variable Measurement

Data collection consisted of electronically collected patient-reported measures and objective use data collected from the VR devices and audio access logs.

In accordance with the Initiative on Methods, Measurement, and Pain Assessment in Clinical Trials (IMMPACT) recommendations, we included multiple methods to evaluate the importance of change in outcome measures across 4 recommended domains: pain intensity, health-related quality of life as defined by physical functioning, emotional functioning, and ratings of overall improvement [41,42].

### Data Collection Time Points

Data were collected across 4 phases of the study: screening, pretreatment baseline, treatment, and posttreatment (day 22). The pretreatment baseline assessment period involved 3 survey time points: days −9, −6, and −3 (averaged to create a single pretreatment baseline value). Surveys were distributed 7 times during the active treatment period (days 1, 3, 6, 9, 12, 15, and 18) and posttreatment on days 21 and 22.

### Measures

The Defense and Veterans Pain Rating Scale (DVPRS) [43] was used to measure average pain intensity, and the DVPRS interference scale was used to measure pain interference on activity, sleep, mood, and stress over the past 24 hours [44].

#### Average Pain Intensity

The average pain intensity was rated for the previous 24 hours using an 11-point numeric rating scale (0=no pain and 10=as bad as it could be; nothing else matters). The average pain intensity was assessed pretreatment (baseline), during treatment, and at the end of treatment on day 21.

#### Pain Interference on Activity, Mood, Sleep, and Stress

Participants were asked to rate the extent to which their pain interfered with their activity, mood, sleep, and stress over the past 24 hours (0=does not interfere and 10=completely interferes). Pain interference was assessed pretreatment (baseline), during treatment, and at the end of treatment on day 21.

#### Pain Catastrophizing

The 13-item Pain Catastrophizing Scale (PCS) [45] is a validated instrument widely used clinically and in pain research to assess patterns of negative cognition and emotion in the context of actual or anticipated pain. Despite having discrete subscales for rumination, magnification, and feelings of helplessness related to pain, prior work has shown that the PCS operates unidimensionally [46]. For this study and the purpose of brevity, the following 4 items were used: “It’s terrible and I think it’s never going to get any better,” “I become afraid that the pain will get worse,” “I can’t seem to keep it out of my mind,” and “I keep thinking about how badly I want the pain to stop.” Respondents rate the frequency in which they experience such thoughts on a scale ranging from 0 (not at all) to 4 (all the time). The items are summed to create a total score and index for pain catastrophizing. The four-item PCS was administered on day 0 (baseline) and on day 22.

#### Pain Self-Efficacy

The two-item Pain Self-Efficacy Questionnaire (PSEQ-2) is a validated instrument used to assess patients’ confidence in their ability to carry out their daily activities [47]. The scale includes the following 2 items: “I can still accomplish most of my goals in life, despite the pain” and “I can live a normal lifestyle, despite the pain.” Respondents rate their responses to the items using a 7-point scale, ranging from 0 (not at all confident) to 4 (completely confident). The two-item scores are summed to create a total score. PSEQ-2 was administered on day 0 (baseline) and on day 22.

#### Patient Global Impression of Change

Aligning with IMMPACT recommendations for pain research [48], Patient Global Impression of Change was assessed on day 22 (posttreatment survey) using the question, “Since the beginning of the study, how has your pain changed?” on a 7-point scale, ranging from *much worse* to *much better*.

#### Satisfaction With Treatment

Satisfaction with treatment was assessed on day 22 (posttreatment survey) using the question, “How satisfied or dissatisfied are you with the ability of this VR (audio) program to relieve your symptoms?” on a 5-point scale (1=extremely dissatisfied to 5=extremely satisfied).

#### Motion Sickness and Nausea

Adverse experiences with using VR were assessed on day 22 (posttreatment survey) using the question, “Did you experience any motion sickness or nausea while using VR?” on a 4-point scale, with 0=never, 1=sometimes, 2=often, and 3=always. This single-item evaluation of cybersickness was adapted from related work on visual images and motion sickness.

### Study Sample and Analytic Data Set

A convenience sample of 97 participants who met all study criteria was enrolled, randomized, and allocated to the VR or audio treatment groups (VR=47 and audio=50). Of 97 participants, 88 (VR=42 and audio=46) provided pretreatment baseline values for the 5 pain indicators and moved to the intervention phase of the study. Of the 88 participants, 74 (VR=35 and audio=39) completed at least one survey over the 21-day treatment phase. The analytic sample comprised 74 participants who completed the baseline measures and at least one survey during the intervention phase.

### Statistical Analysis

The feasibility of the VR treatment was indexed using 3 aspects of the participants’ experience: engagement, satisfaction, and adverse effects nausea/discomfort. For engagement, individual-level session data exist only for the VR group; as such, we presented a descriptive statistic of the total number of sessions launched by the VR and audio groups. Group differences in posttreatment satisfaction (day 22) were assessed using a *t* test. For nausea and discomfort, we provided the proportion of participants who did not experience any nausea/discomfort.

With regard to the secondary outcome of the efficacy of the VR treatment on the 5 pain-related indicators (average pain intensity and pain interference with activity, mood, sleep, and stress), we specified a repeated measures model with time of measurement (henceforth *time*) as the sole predictor. The effect of interest was whether there was a significant improvement in the 5 pain indicators from baseline through the end of treatment, assessed through the significance of the main effect for time. To test the immersive effects of VR relative to audio treatment, we analyzed each of the 5 pain indicators in a linear mixed model framework in which treatment (VR versus audio) was specified as a between-participants factor and time was specified as a within-participants factor with participant-specific intercept and time specified as random effects. The effect of interest was whether the improvement in the pain indicators was different for the VR versus the audio group over time, which was assessed through the significance of the group×time interaction. Posttreatment effect sizes (baseline to treatment completion at day 21) were computed by treatment group using an adaptation of Cohen *d* to suit the repeated measures design [49].

The four-item PCS and two-item PSEQ scales were analyzed in a mixed modeling framework, except that there were only 2 time points (baseline day 0 and day 22). We aimed to test 3 questions: (1) whether pain catastrophizing reduced over time and pain self-efficacy increased over time, both assessed through the significance of the time main effect; (2) whether the VR versus audio group had a differential effect, assessed through the group main effect; and (3) whether the immersive aspect of VR produced a differential impact compared with audio over time, assessed through a time×group interaction.

Group equivalence was assessed through univariate tests of association between treatment groups (VR/audio) on demographics (age, gender, race, household income, number of children ≥17 in the home, employment status, and relationship status), anhedonia, depression, and baseline levels of pain intensity and pain-related interference to sleep, activity, stress, and mood.

## Results

### Sample Characteristics

No significant differences were found between the VR and audio groups for baseline demographic, anhedonia, and depression variables (Table 1). Treatment groups differed in duration of pain since onset, with the VR group having greater pain duration as indexed by the following. Pain duration of 1 year to <5 years represented 40% (14/35) of the VR group and 21% (8/39) of the audio group, whereas pain duration of <1 year represented 3% (1/35) of the VR group and 21% (8/39) of the audio group (*P=*.03).

Furthermore, the baseline values for average pain intensity and pain-related interference with activity, mood, sleep, and stress were found to be equivalent between the study groups (all *P* values>.29; Multimedia Appendix 3).

**Table 1:** Participant characteristics by treatment group (N=74).

Variable		Audio (n=39)	Virtual reality (n=35)	*P* value^a^	
**Gender, n (%)**					**.47**
	Male	26 (67)	26 (74)		
	Female	13 (33)	9 (26)		
**Age group (years), n (%)**					**.63**
	25-34	3 (8)	3 (9)		
	35-44	8 (21)	5 (14)		
	45-54	12 (31)	11 (31)		
	55-64	7 (18)	11 (31)		
	65-74	9 (23)	5 (14)		
**Race, n (%)**					**.63**
	Missing	2 (5)	6 (17)		
	African American	3 (8)	4 (14)		
	Asian	2 (5)	0 (0)		
	White	28 (76)	21 (72)		
	Hispanic/Latino	2 (5)	3 (10)		
	Multiracial/other	1 (3)	0 (0)		
	Native American/Pacific Islander	1 (3)	1 (3)		
**Education, n (%)**					**.17**
	Missing	2 (5)	6 (17)		
	Some high school	3 (8)	0 (0)		
	High school graduate	11 (30)	14 (48)		
	Some college	2 (5)	4 (14)		
	Bachelor degree	13 (35)	6 (21)		
	Postgraduate	8 (22)	5 (17)		
**Employment, n (%)**					**.75**
	Missing	2 (5)	6 (17)		
	Full time	16 (43)	11 (38)		
	Part time	7 (19)	9 (31)		
	Not working	3 (8)	3 (10)		
	Retired	1 (3)	1 (3)		
	Unable to work	10 (27)	5 (17)		
**Marital status, n (%)**					**.16**
	Missing	2 (5)	6 (17)		
	Married/civil union	16 (43)	15 (52)		
	Widowed	1 (3)	1 (3)		
	Divorced/separated	5 (14)	5 (17)		
	Single/cohabitating	1 (3)	4 (14)		
	Single	14 (38)	4 (14)		
**Pain onset, n (%)**					**.03**
	6 months to <1 year	8 (21)	1 (3)		
	1 year to <5 years	8 (21)	14 (40)		
	5 years to <10 years	10 (26)	13 (37)		
	>10 years	13 (33)	7 (20)		
**Anhedonia**					**.20**
	Mean (SD)	1.4 (0.8)	1.1 (0.8)		
	Minimum to maximum	0.0-3.0	0.0-3.0		
	Median (IQR)	1.0 (1.0-2)	1.0 (1.0-2)		
**Depression**					**.15**
	Mean (SD)	1.2 (0.7)	0.9 (0.7)		
	Minimum to maximum	0.0-3.0	0.0-3.0		
	Median (IQR)	1.0 (1.0-2)	1.0 (0.0-1)		

^a^The *P* values represent the parametric test of significant differences between the virtual reality and audio conditions [50].

### Feasibility of At-Home Virtual Reality Interventions for Chronic Pain

This study was designed to provide indicators for feasibility (indexed by participant engagement, satisfaction, and adverse effects) for an at-home self-administered 3-week VR chronic pain treatment program. In the following section, we reported the results for these 3 feasibility indicators.

#### Participant Engagement (Number of Treatment Sessions Completed)

Participants were encouraged to follow the 21-day treatment schedule and told they could repeat any completed experiences at any time. In contrast to the VR group, individual-level session launch data do not exist for the audio group, and engagement data exist only at the group level. As such, we limited the description to the total number of sessions launched by the treatment group for the study period. We observed a total of 1048 and 1067 sessions launched in the audio group and the VR group, respectively (these group-level data do not account for discrepancy in group size). VR participants completed an average of 34.4 sessions (SD 20.30), which exceeded the minimum number (21 sessions) they were asked to complete. Overall, 54 participants responded to the day 22 survey (audio=29 and VR=25), which included items on satisfaction with treatment and motion sickness and nausea (for the VR group).

#### Treatment Satisfaction

Among participants who completed the postintervention survey (n=54), 84% (n=21/25) of participants in the VR group and 72% (n=21/29) of participants in the audio group were either *extremely satisfied* or *very satisfied*.

#### Motion Sickness and Nausea

Of the 25 VR respondents who completed the day 22 survey, 76% (n=19/25) reported never experiencing nausea or motion sickness. Of the 6 participants who reported nausea or motion sickness, 5 reported experiencing the lowest possible symptom frequency (*sometimes*) and 1 participant reported experiencing it *often*. We tested whether motion sickness and nausea impacted the engagement with the VR treatment by examining the number of VR sessions launched for those with *sometimes* (n=5) and *often* (n=1) nausea/motion sickness and the remainder of the VR group (n=29). We found that experiencing nausea/motion sickness at the lowest level did not negatively impact the use of VR, as indexed by the number of sessions launched (36 sessions versus 33 sessions for the remainder of the VR group). However, the single individual who reported nausea/motion sickness *often* launched only one-third of the number of sessions compared with the remainder of the VR group (11 sessions versus 33 sessions).

### Survey Completion

There was no group difference for completion of the 8 treatment surveys administered during and immediately following the 21-day treatment period (VR=5.5 and audio=5.6; *P=*.89).

### Preliminary Efficacy on Patient-Reported Outcomes (Virtual Reality Group Only)

For the VR group, we found significant effects on each of the 5 pain indicators using the repeated measures model in which time was the sole predictor (all *P*<.001). For brevity and clarity, we included only the baseline and 21-day mean values in the text but provided the mean values for all time points in the figures. The average reductions from baseline through day 21 were as follows: pain intensity was reduced by 30% (mean reduced from 4.6 to 3.2; Cohen *d* effect size of 0.71), pain-related activity interference reduced by 37% (mean reduced from 4.9 to 3.1; Cohen *d* effect size of 0.83), pain-related mood interference reduced by 50.0% (mean reduced from 5.4 to 2.7; Cohen *d* effect size of 0.94), pain-related sleep interference reduced by 40.4% (mean reduced from 5.2 to 3.1; Cohen *d* effect size of 0.87), and pain-related stress interference reduced by 49.1% (mean reduced from 5.3 to 2.7; Cohen *d* effect size of 0.89). All the aforementioned effect sizes met or exceeded the >30% threshold for clinically important changes, and improvement in pain-related mood interference met the substantial clinical importance threshold of >50% [48,51].

### Comparison Between the Virtual Reality and Audio Treatment Groups

**Figure 4:**
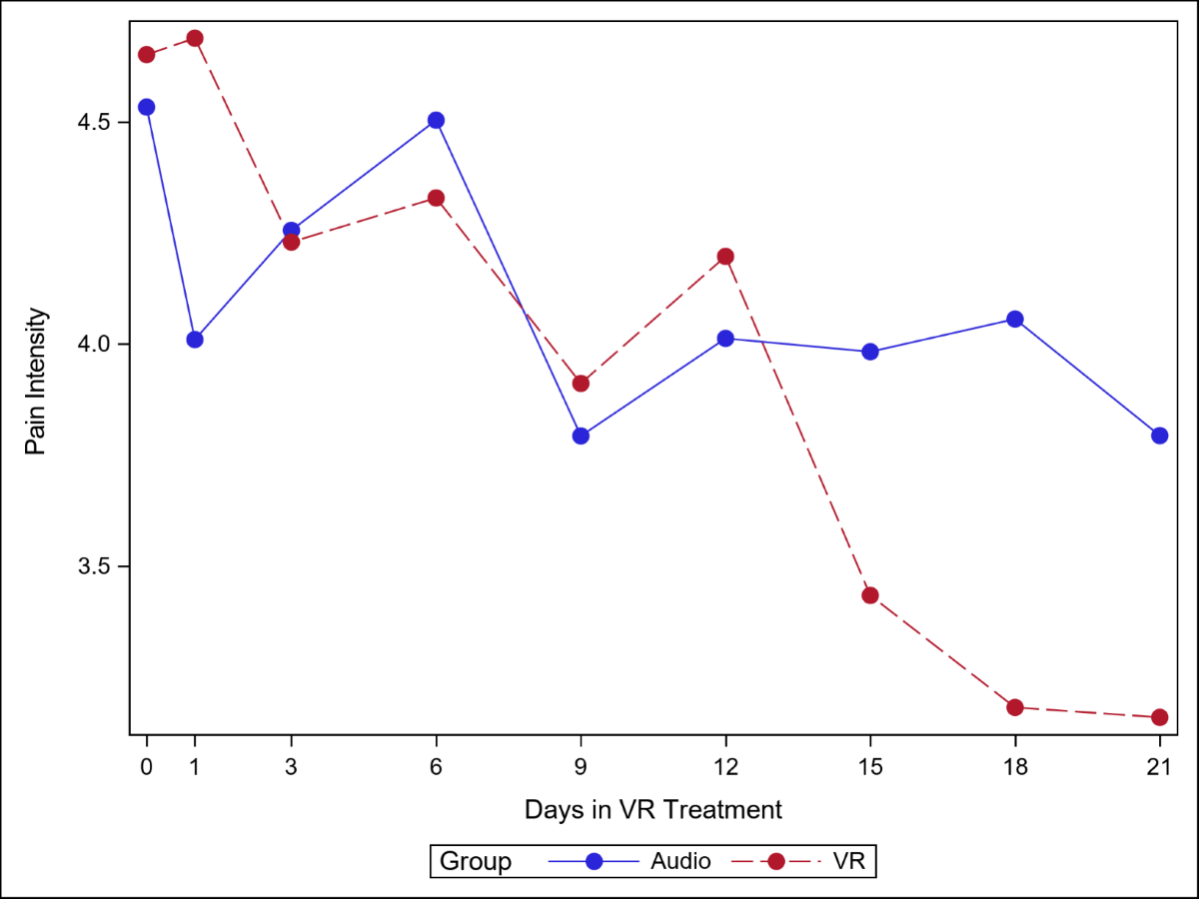
Effect of virtual reality vs audio on pain intensity over time.

**Figure 5:**
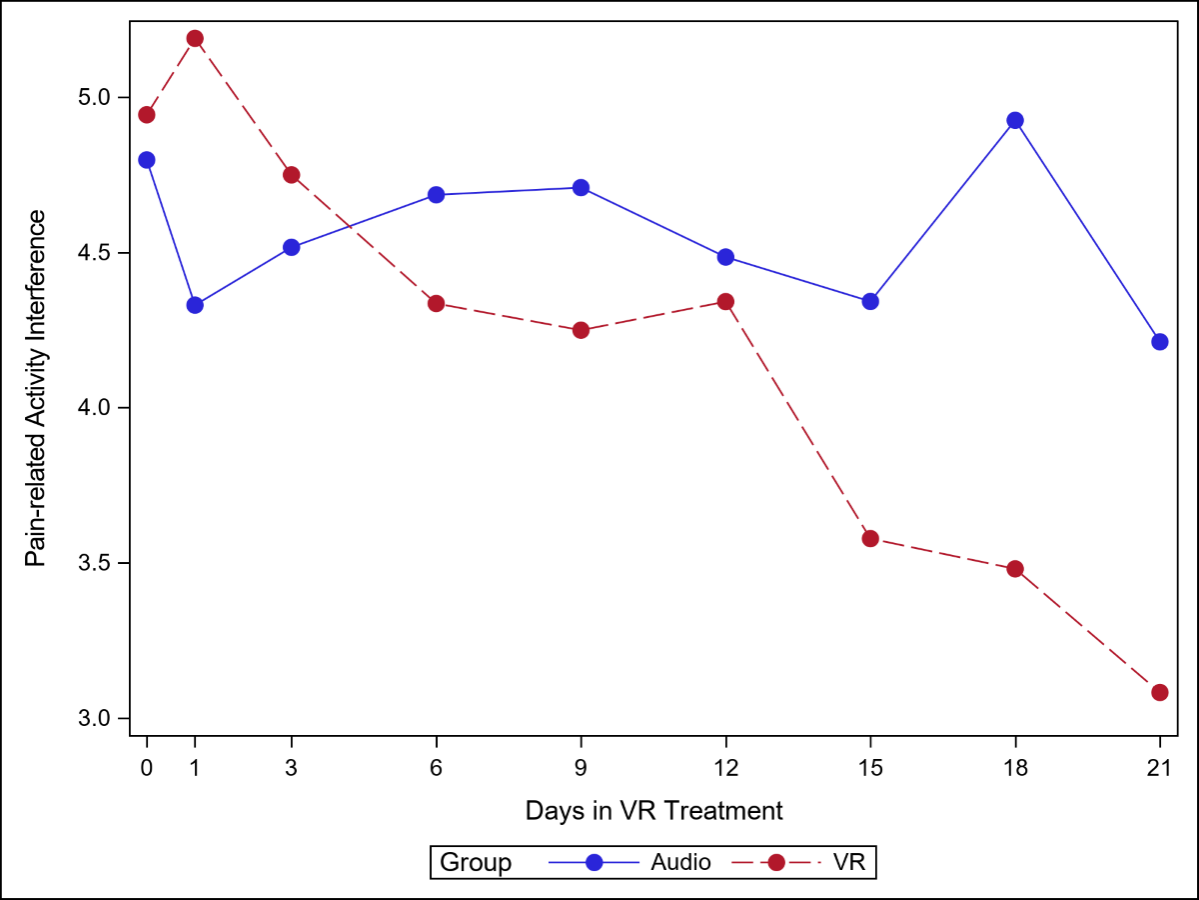
Effect of virtual reality vs audio on pain-related activity interference over time.

**Figure 6:**
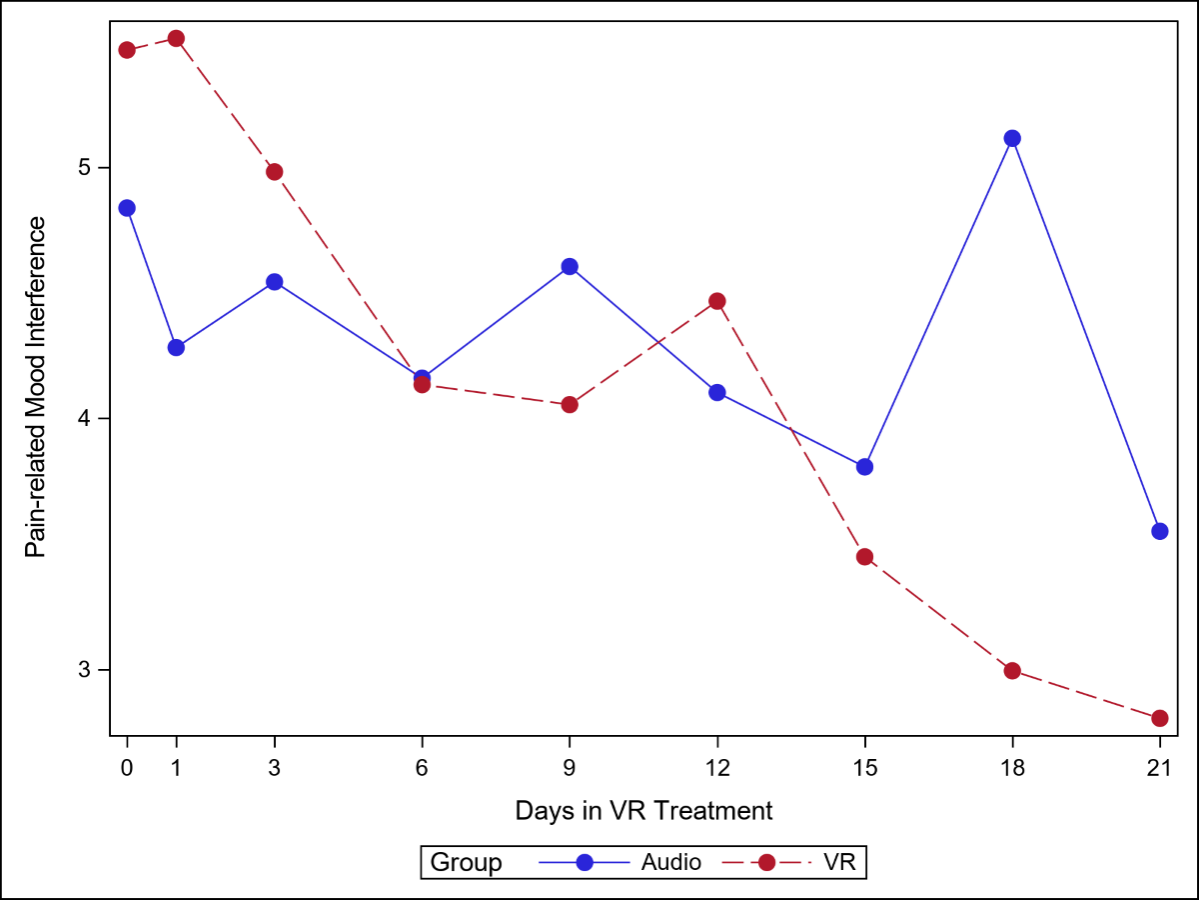
Effect of virtual reality vs audio on pain-related mood interference over time.

**Figure 7:**
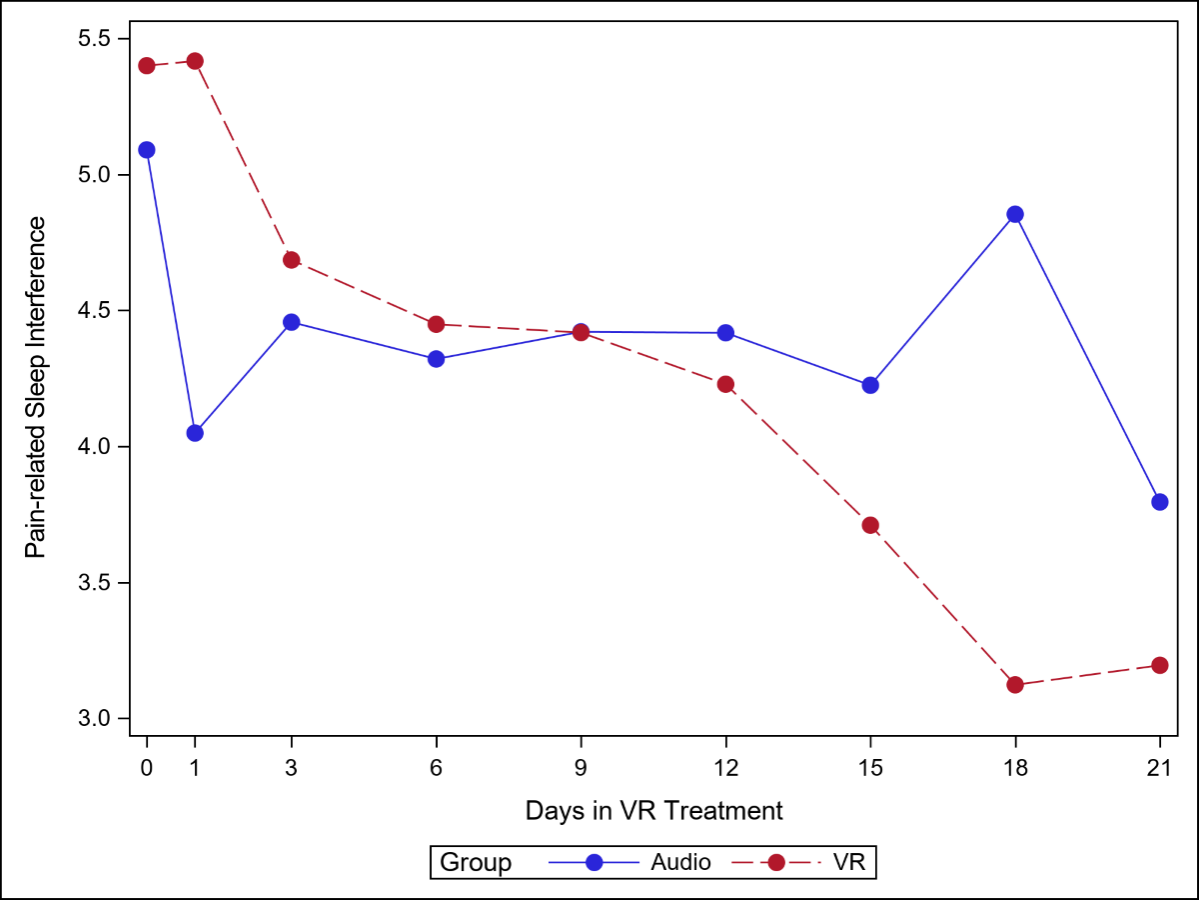
Effect of virtual reality vs audio on pain-related sleep interference over time.

**Figure 8:**
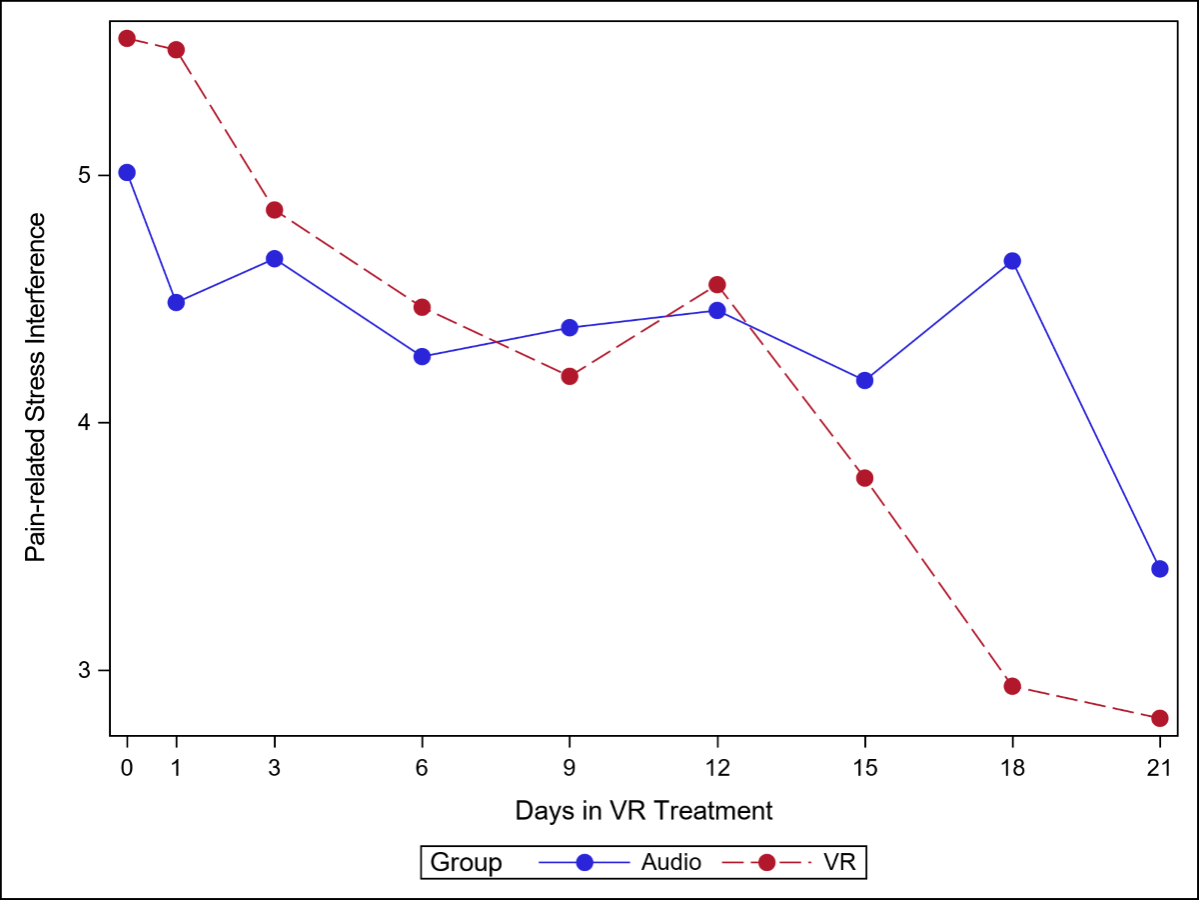
Effect of virtual reality vs audio on pain-related stress interference over time.

The 5 figures below (Figures 4-8) compared group effects over time for the 5 pain indicators. For all figures, the trend of the pain-related variable was displayed over time for participants in the VR and audio groups. Values in the x-axis refer to the number of days in the study, where *0* represents the baseline computed as the average of the 3 preexposure measures on −9, −6, and −3 days before the start of the treatment.

#### Average Pain Intensity

Pain intensity decreased over time in both groups, and the decline was steeper for the VR and audio groups (*P=*.04), with differences becoming more pronounced from day 15 onward. It should be noted that none of the simple effects (ie, effect of group within time slice) are significant (Figure 4). As seen in Multimedia Appendix 4, from baseline to day 21, consistent with the time×group interaction, the Cohen *d* was smaller in the audio group than in the VR group (0.42 and 0.71, respectively).

#### Pain-Related Activity Interference

Activity interference decreased more steeply in the VR group than in the audio group (*P=*.005), with differences becoming more pronounced from day 15 onward (Figure 5). The simple effect (ie, effect of group within time slice) was significant at day 18 (VR<audio; *P=*.02). As seen in Multimedia Appendix 4, Cohen *d* was smaller in the audio group than in the VR group (0.26 and 0.83, respectively).

#### Pain-Related Mood Interference

As shown in Figure 6, mood interference appeared to decrease more steeply in the VR group than in the audio group. The difference between the VR and audio groups became more pronounced from day 15 onward. The simple effect (ie, effect of group within time slice) was significant at day 18 (VR<audio; *P<*.001). As seen in Multimedia Appendix 4, Cohen *d* was smaller in the audio group than in the VR group (0.76 and 0.94, respectively).

#### Pain-Related Sleep Interference

Reductions in sleep interference were greater in the VR group, with the simple effects (ie, effect of group within time slice) reaching significance at day 1 (audio<VR; *P=*.02) and on day 18 (VR<audio, Figure 7; *P=*.002).

As seen in Multimedia Appendix 4, Cohen *d* was smaller in the audio group than in the VR group (0.64 and 0.87, respectively).

#### Pain-Related Stress Interference

As shown in Figure 8, stress interference appeared to decrease more steeply in the VR group compared with the audio group, and this difference was more pronounced from day 15 onward. The simple effect (ie, effect of group within time slice) was significant on day 18 (*P=*.01). As seen in Multimedia Appendix 4, Cohen *d* was smaller in the audio group than in the VR group (0.87 and 0.89, respectively).

#### Pain Catastrophizing

We observed a significant main effect of time, with pain catastrophizing decreasing for both groups over time (*P<*.001). The main effect for group was not significant (*P=*.61). Finally, we did not perform a time×group interaction (*P*=.52).

#### Pain Self-Efficacy

We observed a significant main effect of time, with pain self-efficacy increasing in both groups over time (*P<*.047). The main effect for group was not significant (*P=*.68). Finally, we did not perform a time×group interaction (*P*=.46).

#### Global Impression of Change

At day 22, among survey responders (n=54), 84% (n=21/25) of participants in the VR group reported that their pain was improved, 16% (n=4/25) reported no change, and 0% (n=0/25) reported worsening pain. In the audio group, 62% (n=18/29) of participants reported improvement, 34% (n=10/29) reported no change, and 3% (n=1/29) reported worsening pain.

Finally, because of the observed group difference in the duration of chronic pain (indexed as pain onset in Table 1), we conducted additional analyses with pain onset specified as a covariate in the model with time, group, and time×group as predictors of the 5 pain variables. The significance of the time main effect and time×group interaction effects was fully preserved. When intention-to-treat (ITT) analyses were applied all of the results were preserved with one exception; the time×treatment interaction was no longer significant.

## Discussion

### Principal Findings

We conducted an unblinded randomized controlled study in a web-based convenience sample of community-based participants with nonmalignant chronic low back pain and/or fibromyalgia by comparing at-home self-administered VR treatment with an audio-only treatment group (same audio content as VR with minor modifications made for one-third of the modules). The primary goal of the study was to evaluate the feasibility of a self-administered home-based VR program for chronic pain that included skills-based content informed by evidence-based CBT for chronic pain. The secondary goal was to conduct an RCT of the VR treatment to an audio-only treatment. We aimed to determine the preliminary efficacy of VR for reducing average pain intensity and pain-related interference with activity, mood, sleep, and stress over the 21-day treatment program. Our tertiary goal was to isolate the immersive effects of the skills-based VR program by comparing the effects between treatment groups.

VR demonstrated good feasibility as indexed by excellent participant engagement (average of 34 sessions completed over the 21-day treatment program), high ratings for satisfaction with the treatment (84%), and relatively low reporting for motion sickness and nausea (n=6/25, 24% of VR participants who completed the day 22 survey). Of these 6 participants, 5 reported the lowest level of symptom frequency possible (*sometimes*). We found that this symptom level did not interfere with VR treatment engagement, as indexed by the number of sessions launched compared with participants in the remainder of the VR group. The single individual who experienced nausea and motion sickness *often* showed markedly decreased use of VR (11 sessions launched versus 34 for people with low nausea and the remainder of the VR group).

With respect to preliminary efficacy, VR demonstrated the significant reduction in average pain intensity and pain-related interference in activity, mood, sleep, and stress over the course of the 21-day treatment program. Treatment effect sizes suggested that a home-based stand-alone, digital, skills-based treatment program may affect clinically meaningful changes in patient-reported pain and pain correlates. The durability of the treatment effects reported here remains a topic for future research.

Although a significant body of research exists on the use of VR for acute pain management [26,28,30,52-54] and for physical rehabilitation [24,40], the use of VR as a platform to deliver behavioral medicine for chronic pain remains novel and understudied. Research on VR for acute pain is based on the premise that distraction is a primary mechanism of VR analgesia [33,55]. Therefore, the effects of distraction on pain are typically measured within a rapid time frame using study designs that align with drug trials. Investigations of VR for chronic pain require a different approach to align with the goal of sustained pain management. Although VR for physical rehabilitation shows sustained results, content is typically constrained to rehabilitative movement, exercise, and kinematic training [24,40] and is devoid of didactics and skills training contained in evidence-based behavioral medicine treatments [7].

Unlike distraction alone, behavioral health therapies rarely produce instantaneous results. Rather, skills acquisition and mastery require time, in part because of the multisession delivery of content, and become effective over the course of weeks, as the content is delivered more comprehensively, and patients practice skills during and between sessions and is correlated with patient engagement [56]. Our prior work suggests that an ultrabrief skill-based intervention for chronic pain evidenced clinically meaningful improvements in pain-related symptoms at 2 weeks, with even greater improvements evidenced at 4 weeks [12]. The results presented here dovetail with this literature and our fundamental understanding of how didactic and skills-based behavioral medicine treatment results accrue over time as participants receiving increasing amounts of knowledge and skills practice during active treatment [6,56,57]. In this study, the emergence of a more pronounced improvement in pain outcomes at day 15 supports the hypothesis that the didactic and skills-based elements of immersive behavioral medicine VR are operating as expected, although confirmatory studies are needed.

In evaluating the method of delivery of a skills-based treatment program, this study demonstrated superior reduction of most pain indicators in the VR group relative to the audio group after 2 weeks. Treatment group differences remained minimal until day 12, with VR superiority strengthening from day 15 onward and peaking on day 18. For the pain interference outcomes, the VR group showed greater improvement compared with the audio group. The exceptions were pain catastrophizing and pain self-efficacy, which improved for both treatment groups. We have no evidence that VR’s superior treatment effects were explained by user engagement, as we observed similar rates of engagement for both groups. Our study design allowed us to isolate the immersive effect of VR relative to active treatment delivered via audio format only. On balance, our findings suggest that the treatment effect sizes for VR are both statistically significant and clinically meaningful for pain intensity and across the pain interference variables and are superior to the same treatment delivered by audio alone. A key aspect of the immersive VR experience involves dynamic interaction between the user’s breath and the environment, wherein voice-over coaching directs the user to slow the breath to engage a parasympathetic response. The environment responds to the breath, provides visual feedback to the user, and possibly affords users enhanced acquisition of this skill relative to the audio-only content.

### Strengths and Limitations

The strengths and limitations of our study bear careful attention. The interpretation of study findings is limited by the 2 pain conditions studied and the selection bias inherent in the web-based convenience sample. In addition, the analytic data set included only those participants who completed at least one study survey; accordingly, larger studies are needed to confirm the findings reported here and to determine generalizability. Medication use was not assessed and may have been a confounding variable. Chronic pain type and duration were self-reported, and there was no review of medical records to confirm diagnoses. Our ability to assess VR satisfaction and nausea/motion sickness was limited by only 25 of 35 participants completing the day 22 posttreatment survey. Although only 17% of the full VR sample reported experiencing cybersickness to any degree, we cannot rule out the possibility that early attrition may be partially attributable to these adverse effects, although notably, we did not find disparate attrition rates between the VR and audio groups. Commercially available VR programs typically offer a money-back guarantee trial period to allow customers to return their VR device for refund in cases of cybersickness. Although assignment to treatment group was random, differences in duration of pain since onset between the 2 treatment groups may have influenced pain outcomes during the course of the study, and we note that this difference favored the audio group. In addition, the analysis did not focus on the correlation between individual-level variations in the use of the intervention (VR or audio) and the pain indicators, and this remains a topic for future study.

Our study design merits consideration within the context of our findings. The audio treatment group was an active comparator with two-thirds of the audio content identical to the audio of the VR group, and one-third of the audio content closely matched the VR content for topical content, skills, and experience (minus the visual and interactive elements). This study was rigorously designed to isolate the immersive effects of VR rather than simply comparing VR with placebo or with a weaker control group such as *usual care*. In studying a treatment delivered 2 ways, our results suggest that the immersive (3D visual elements, dynamic scenery, and 360-degree vision capability) and user interactivity with the environment are pleasing, engaging, and generally effective for those who do not experience cybersickness (many commercial VR companies offer consumers a trial period or money-back guarantee for cases of cybersickness).

Finally, the cost-effectiveness of VR for chronic pain relative to in-person behavioral medicine visits or other digital treatment options merits investigation. In-person behavioral medicine treatments require multiple clinic visits, travel costs, time from work and other obligations, and treatment copayment costs that may total several hundreds of dollars over a standard 8-session treatment package. Future research should compare home-based VR to in-person multisession CBT for chronic pain in terms of efficacy and cost-effectiveness. Although some 2D digital treatment options, such as web-based pain CBT or self-management programs, and may be sourced at no cost, engagement rates remain relatively low. Future research may explore participants’ perceived comparative value of VR to audio-only treatment. VR provides patients and clinicians with a new home-based treatment option that may be preferred by some patients and also provide more effective pain management to a subset of individuals. Additional studies of longer duration may investigate the durability of treatment effects reported here.

### Conclusions

This study is one of the first to explore how a self-administered home-based VR program rooted in behavioral medicine skills and techniques impacts chronic pain. The findings broadly suggest that VR holds promise as effective, stand-alone, home-based digital behavioral medicine for chronic pain. Additional studies are needed, including larger sample sizes, diverse chronic pain conditions, longer duration of study to best characterize the efficacy of VR in chronic pain management, and the impact of VR on other factors such as pain medication use and medical care utilization. Future studies may further elucidate the VR mechanisms of action and VR’s role in expanding access to multimodal behavioral medicine for chronic pain.

## Appendix

Multimedia Appendix 1 Participant consent form.

Multimedia Appendix 2 Schedule of 21-day virtual reality and audio programs.

Multimedia Appendix 3 Baseline pain-related variables by treatment group.

Multimedia Appendix 4 Effect sizes for change from baseline to day 21.

## Abbreviations

CBT: cognitive behavioral therapy
DVPRS: Defense and Veterans Pain Rating Scale
IMMPACT: Initiative on Methods, Measurement, and Pain Assessment in Clinical Trials
PCS: Pain Catastrophizing Scale
PSEQ-2: two-item Pain Self-Efficacy Questionnaire
RCT: randomized controlled trial
VR: virtual reality
