# Supplementary material for: Self-Administered Skills-Based Virtual Reality Intervention for Chronic Pain: Randomized Controlled Pilot Study
Source: JMIR Form Res. 2020 Jul 7;4(7):e17293. doi: 10.2196/17293 (PMC7381022; doi:10.2196/17293)
Supplement: Multimedia Appendix 1 [file formative_v4i7e17293_app1.doc]

**RESEARCH SUBJECT INFORMATION AND CONSENT FORM**

**TITLE:** Virtual Reality for At-Home Chronic Pain Self-Management Pilot Study

**PROTOCOL NO.:** None

WIRB® Protocol #20190248

**Sponsor:** appliedVR, Inc.

**INVESTIGATOR:** Dr. Jorge Minor, MD

1840 Century Park East, 8^th^ floor

Century City, CA 90067

United States

**PHONE NUMBER(S):** Jeannette Tsuei

**844-204-9093** (24 Hours) or 911 for emergency

424-218-6470

Dr. Jorge Minor, MD

213-482-1046

**WHAT IS THE PURPOSE OF THE STUDY?**

You are invited to participate in a study being conducted by appliedVR. The purpose of the study is to evaluate the effectiveness of a 21-day digital health wellness program in managing chronic pain and improving quality of life with pain. Some subjects will experience the digital health program using a virtual reality headset. Other subjects will listen to the digital health program using audio recordings. The virtual reality device is experimental for pain treatments. The device and software pose minimal risks to subjects.

**WHAT WILL HAPPEN DURING THE STUDY?**

After you agree to participate in the study, you will be asked to complete surveys every 2-3 days to measure your pain levels. appliedVR will provide you with the digital health program and ask you to complete one session each day for 21 days. Each session will last between 3-15 minutes.

The Investigator will randomly select the type of digital health program for you. If you are assigned to the virtual reality group, you will be asked to wear a device, the Oculus Go, with preloaded visualization software which will allow you to experience being part of a three-dimensional world that is intended to be pleasant and appears quite real. If you are assigned to the audio recording group, you will be asked to visit the site (https://paincarepilot.squarespace.com/) where you can download or stream the recordings on your smartphone or laptop. You have an equal chance of being assigned to the virtual reality group or the audio recording group.

Prior to starting the digital health program, you will be asked to complete a short online questionnaire which will ask you about your current health. Throughout the duration of the 21-day program, we will ask you to complete very brief online questionnaires every 2-3 days to measure your pain level. At the end of the 21-day program, we will ask you to complete an online questionnaire to understand your experience with the program.

Participation in the study should not take more than 45 days in total.

**RISKS AND DISCOMFORTS**

Virtual Reality Intervention

Patients using the virtual reality intervention may experience side effects common to users of VR and individuals who view 3D video, including motion sickness, dizziness, eye strain, headaches, or other visual abnormalities. If participants experience these symptoms while using the device at home, they should stop using the device for 15 minutes and then attempt to resume use of the device. If the symptoms do not resolve or recur and you are unable to continue the use of VR, you should contact the study team at the phone number listed on the first page. A small number of patients (up to 0.025%) may experience seizures or severe symptoms (e.g., disorientation, nausea, or drowsiness) upon viewing the virtual reality experience. Seizures from flashing light are more common in children and epileptic patients (who are excluded). To minimize this concern further, we have *not* incorporated flashing lights into the VR experiences. Some patients may find the VR goggles uncomfortable to wear or confining. To date, patients with claustrophobia have *not* reported discomfort using VR goggles, as they are often used in treatment of that condition. Nevertheless, individuals previously diagnosed with claustrophobia should discontinue use if they feel uncomfortable.

Audio Recordings Intervention:

There are no physical risks and listening to the audio recordings is not uncomfortable.

Questionnaires:

It is possible that some of the items in the questionnaire may make you feel uncomfortable or embarrassed. Specifically, some items ask you about your overall mental health, irritability, and anxiety. You are not required to respond to any item that you do not wish to answer. We will not use your responses to diagnose any mental illness, but your responses may prompt a member of the study staff to follow up with your doctor. For example, if you report that you “often” feel like you need help with your depression, we will notify your provider to contact you about this issue. You are not required to pursue any care recommendations that stem from this interaction. Further, your responses will not affect current or future care received from your provider.

There is the possible risk of loss of confidentiality of your research information.

**BENEFITS**

If you agree to take part in this research study, there may or may not be direct medical benefit to you. The possible benefits of taking part in the research study are greater care satisfaction, pain management, and overall health during the study. However, no benefit is guaranteed. It is possible that your condition may remain unchanged or even get worse.

We hope the information learned from this research study will benefit other patients in the future by helping us to learn whether patients enjoy using the VR interventions. Knowledge gained could be used to improve pain management across chronic pain sufferers in the future.

# **WHY WOULD MY PARTICIPATION BE STOPPED?**

Your participation in this study may be stopped at any time by the researcher or the sponsor without your consent for any reason, including:

- The study is stopped or suspended;
- Support for the study is reduced, stopped or withdrawn;
- If it is in your best interest;
- You do not consent to continue in the study after being told of changes in the research that may affect you;
- You become pregnant; or
- You do not follow the study procedures.

# **ARE THERE ANY OTHER OPTIONS?**

Other options for chronic pain relief include medication, cognitive behavior therapy, physical therapy, and relaxation techniques.

Your participation is voluntary so you have the right to decline to participate or to withdraw from this research study at any time without any penalty or loss of benefits to which you would otherwise be entitled outside of the study.

# **WILL MY INFORMATION BE KEPT CONFIDENTIAL?**

Your survey answers will be stored initially with REDCap Cloud Electronic Data Collection (EDC) in a password protected electronic format. Data will later be downloaded and stored in a password-protected and encrypted Excel database which will have a subject number and initials and not have any personal identifiers associated with the survey responses.

We will do our best to make sure that your personal information collected as part of this study is kept private. However, we cannot guarantee total privacy. Your personal information may be given out if required by law. If information from this study is published or presented at scientific meetings, your name and other identifiable personal information will not be used. Organizations that may look at and/or copy your survey responses for research, quality assurance, and data analysis include: the Institutional Review Board, accrediting agencies, government and regulatory groups (such as Food and Drug Administration (FDA), Office for Human Research Protections (OHRP), etc.), safety monitors, companies that sponsor the study, and authorized representatives of the sponsor.

# **WHAT IF I BECOME ILL OR INJURED BECAUSE OF TAKING PART IN THIS STUDY?**

Should you believe that you are ill or have been injured as a result of your participation, please contact the study team at the phone number listed on page 1 of this consent form.

# **FINANCIAL CONSIDERATIONS**

*Costs of Participation*

You will not be charged for your participation in this research study. The study sponsors will cover the cost of all items and services required by this study. You will be asked to return the virtual reality kit to appliedVR at completion of the study and be given a pre-paid shipping label to mail the kit back to appliedVR.

Compensation for Participating

You will receive $30 USD after completing the 21-day program and the post-intervention survey administered at the end of the study. If you do not complete the study, you will be given a prorated amount based on the number of days completed. For example, if you complete 5 of the 21 sessions, you will receive a $7 Amazon.com gift card.

# **WHAT IF I HAVE QUESTIONS OR PROBLEMS?**

Please contact one of the investigators listed on the first page of this form if you have a research-related problem or if you have questions, complaints, or concerns about the research.

If you have questions about your rights as a research participant, general questions, complaints, problems, or concerns that you want to discuss with someone who is not associated with this study, or want to offer suggestions or feedback, please contact:

Western Institutional Review Board® (WIRB®)

1019 39th Avenue SE Suite 120

Puyallup, Washington 98374-2115

Telephone: 1-800-562-4789 or 360-252-2500

E-mail: Help@wirb.com

WIRB is a group of people who independently review research.

WIRB will not be able to answer some study-specific questions, such as questions about appointment times. However, you may contact WIRB if the research staff cannot be reached or if you wish to talk to someone other than the research staff.

# **ELECTRONIC CONSENT**

If you sign this form below, it indicates that:

- You have taken the time to carefully read and understand the information presented in this informed consent form;
- You have considered the potential risk and any anticipated benefits of participation as described in this consent form;
- You voluntarily agree to participate in this research study;
- You are 18 years of age or older

 Agree

 Disagree

NAME:

ELECTRONIC SIGNATURE VIA REDCAP CLOUD ECONSENT:

DATE:

We will email you an electronic copy of this signed and dated consent form to keep for your records.
