# Supplementary material for: Self-Administered Skills-Based Virtual Reality Intervention for Chronic Pain: Randomized Controlled Pilot Study
Source: JMIR Form Res. 2020 Jul 7;4(7):e17293. doi: 10.2196/17293 (PMC7381022; doi:10.2196/17293)
Supplement: Multimedia Appendix 2 [file formative_v4i7e17293_app2.docx]

**Multimedia Appendix 2. Schedule of 21-Day VR and Audio Programs**

(Bolded titles include identical audio for both programs)

|  | **VR Program** | **Audio Only Program** |
| --- | --- | --- |
| **Day 1** | Breath of Hope | Welcome to Mindfulness |
| **Day 2** | Breathing Life | Breathing in Five Senses |
| **Day 3** | **Well-being Relaxation** | **Well-being Relaxation** |
| **Day 4** | Dolphin Healing | Releasing Pain |
| **Day 5** | Focus Game | Body Scan |
| **Day 6** | Paced Breathing | Breathing in Five Senses |
| **Day 7** | **Awareness of the Mind** | **Awareness of the Mind** |
| **Day 8** | **Intro to Pain Education** | **Intro to Pain Education** |
| **Day 9** | Dream Garden | Cosmic Journey |
| **Day 10** | Building Breath | Mindful Breathing |
| **Day 11** | Mallorca | Empower |
| **Day 12** | **Body Scan** | **Body Scan** |
| **Day 13** | Breathing Portal | Releasing Pain |
| **Day 14** | **Compassion Relaxation** | **Compassion Relaxation** |
| **Day 15** | **Mindful Breathing** | **Mindful Breathing** |
| **Day 16** | **Let’s Talk About Pain** | **Let’s Talk About Pain** |
| **Day 17** | Deep Relaxation | Breathing in Five Senses |
| **Day 18** | **Harm Alarm** | **Harm Alarm** |
| **Day 19** | **Empowerment** | **Empowerment** |
| **Day 20** | Dream Maker | Breathing in Five Senses |
| **Day 21** | **Power of the Mind** | **Power of the Mind** |
