# Supplementary material for: Self-Administered Skills-Based Virtual Reality Intervention for Chronic Pain: Randomized Controlled Pilot Study
Source: JMIR Form Res. 2020 Jul 7;4(7):e17293. doi: 10.2196/17293 (PMC7381022; doi:10.2196/17293)
Supplement: Multimedia Appendix 3 [file formative_v4i7e17293_app3.docx]

**Multimedia Appendix 3. Baseline Pain-related Variables by Treatment Group**

| **Measure** | **Audio (N=39)** | **VR (N=35)** | **p-value** |
| --- | --- | --- | --- |
| Pain Self Efficacy |  |  | 0.94 |
| Nmiss (%) | 3 (7.7) | 5 (14.3) |  |
| Mean±SD | 7.3±2.9 | 7.2±2.4 |  |
| Min–Max | 2.0–12.0 | 3.0–12.0 |  |
| Median (IQR) | 8.0 (5.5–9 | 8.0 (5.0–9 |  |
| Pain Catastrophizing |  |  | 0.89 |
| Nmiss (%) | 1 (2.6) | 5 (14.3) |  |
| Mean±SD | 8.5±3.0 | 8.4±3.5 |  |
| Min–Max | 4.0–16.0 | 2.0–16.0 |  |
| Median (IQR) | 8.0 (7.0–1 | 8.5 (6.0–1 |  |
| Average Pain Intensity |  |  | 0.77 |
| Mean±SD | 4.5±1.8 | 4.7±1.7 |  |
| Min–Max | 1.3–8.7 | 1.3–8.0 |  |
| Median (IQR) | 4.7 (3.0–5 | 5.0 (4.0–6 |  |
| Pain Stress Interference |  |  | 0.45 |
| Nmiss (%) | 2 (5.1) |  |  |
| Mean±SD | 5.1±2.6 | 5.6±2.8 |  |
| Min–Max | 1.0–10.0 | 1.0–10.0 |  |
| Median (IQR) | 5.3 (3.0–6 | 7.0 (3.0–7 |  |
| Pain Mood Interference |  |  | 0.29 |
| Mean±SD | 4.8±2.4 | 5.5±2.7 |  |
| Min–Max | 1.0–9.7 | 1.0–10.0 |  |
| Median (IQR) | 5.0 (3.0–6 | 5.7 (3.3–7 |  |
| Pain Sleep Interference |  |  | 0.6 |
| Nmiss (%) | 1 (2.6) | 1 (2.9) |  |
| Mean±SD | 5.2±2.4 | 5.5±2.6 |  |
| Min–Max | 1.0–10.0 | 1.0–9.3 |  |
| Median (IQR) | 4.7 (3.3–7 | 5.4 (3.7–7 |  |
| Pain Activity Interference |  |  | 0.84 |
| Nmiss (%) | 1 (2.6) |  |  |
| Mean±SD | 4.8±2.2 | 4.9±2.1 |  |
| Min–Max | 1.0–9.0 | 1.0–8.0 |  |
| Median (IQR) | 4.7 (3.3–6 | 5.3 (3.7–6 |  |

The p-values represent the parametric test of significant differences between the VR and Audio condition. This table was prepared using output from the %ggbaseline macro for SAS®.
