# Supplementary material for: Self-Administered Skills-Based Virtual Reality Intervention for Chronic Pain: Randomized Controlled Pilot Study
Source: JMIR Form Res. 2020 Jul 7;4(7):e17293. doi: 10.2196/17293 (PMC7381022; doi:10.2196/17293)
Supplement: Multimedia Appendix 4 [file formative_v4i7e17293_app4.docx]

Table 4: Effect Sizes for Change from Baseline to Day 21

| **Variable** | **Condition** | **Mean** | **Std Dev** | **Cohen's dz** |
| --- | --- | --- | --- | --- |
| Pain Average | Audio | 0.76 | 1.79 | 0.42 |
|  | VR | 1.48 | 2.08 | 0.71 |
| Pain Activity Interference | Audio | 0.62 | 2.33 | 0.26 |
|  | VR | 1.95 | 2.35 | 0.83 |
| Pain Mood Interference | Audio | 1.58 | 2.08 | 0.76 |
|  | VR | 2.61 | 2.79 | 0.94 |
| Pain Sleep Interference | Audio | 1.33 | 2.09 | 0.64 |
|  | VR | 2.25 | 2.58 | 0.87 |
| Pain Stress Interference | Audio | 1.67 | 1.92 | 0.87 |
|  | VR | 2.67 | 3.02 | 0.89 |
| The column “Mean” refers to the difference between baseline and day 21 of the variable in question, and “Std Dev” refers to the standard deviation of the difference. Cohen’s dz was computed as the ratio of the mean to standard deviation of the difference variable citation here. | | | | |
